# Early-Onset Signet-Ring Cell Adenocarcinoma of the Colon: A Case Report and Review of the Literature

**DOI:** 10.1155/2017/2832180

**Published:** 2017-02-23

**Authors:** Maliha Khan, Krittiya Korphaisarn, Aneeqa Saif, Wai C. Foo, Scott Kopetz

**Affiliations:** ^1^Department of Leukemia, The University of Texas MD Anderson Cancer Center, Houston, TX, USA; ^2^Department of Gastrointestinal Medical Oncology, The University of Texas MD Anderson Cancer Center, Houston, TX, USA; ^3^Department of Internal Medicine, Dow University of Health Sciences, Karachi, Pakistan; ^4^Department of Pathology, The University of Texas MD Anderson Cancer Center, Houston, TX, USA

## Abstract

Colorectal cancer (CRC) remains the second leading cause of cancer-related deaths in the United States. While a decline has been observed in the older population, the occurrence of CRC in the adolescent and young adult (AYA) population has increased over the past two decades. The histopathologic characteristics and clinical behavior of CRC in AYA patients have been shown to be distinct from those of CRC in older adults. The rarer subtypes of CRC such as mucinous adenocarcinoma and signet-ring cell carcinoma are associated with a poorer prognosis compared to the more common subtypes. Here we report a case of a 20-year-old man who was diagnosed with stage IVB (T4 N2 M1, with peritoneal carcinomatosis) signet-ring cell adenocarcinoma of the colon. The scarcity of information on these rarer subtypes merits further study and investigation.

## 1. Introduction

Colorectal cancer is the third most common cancer worldwide and the second leading cause of cancer-related deaths in the United States. The incidence of CRC in the adolescent and young adult (AYA) population has increased over the past two decades [1] and has recently been highlighted as a common problem in various countries [2–4]. The histopathologic characteristics and clinical behavior of AYA CRC patients have been shown to be distinct from those of CRC in older adults. Chang et al. reported a large series of AYA study to date that early-onset (<40 years) CRC had more frequently either presented with or developed metastatic (45% versus 25%, *P* = 0.014) and predominately sigmoid colon and rectum (*P* < 0.007) [5]. Moreover, early-onset CRC were much more likely to demonstrate adverse histologic factors, including frequency of signet-ring cell differentiation (13% versus 1%), perineural invasion (29% versus 11%, *P* = 0.09), and venous invasion (22 versus 6%) compared with patients > 40 years of age. More recently, CRC patients in the National Cancer Data Base from 1998 to 2011 were grouped into pediatric (</=21 years), early-onset adult (22–50), and older adult (>50) patients [6]. In this cohort, pediatric histology was more likely signet-ring, mucinous, and poorly differentiated. Initial treatment was usually surgery, but patients </=50 were more likely to have radiation (Ped: 15.1%, EA: 18.6%, and OA: 9.2%) and chemotherapy (Ped: 42.0%, EA: 38.2%, and OA: 22.7%). Children and older adults showed poorer overall survival at 5 years when compared to early-onset adults. Adjusting for covariates, age </=21 was a significant predictor of mortality for colon and rectal cancers (colon HR: 1.22, rectal HR: 1.69) [6].

The molecular alteration of early-onset CRC has not been well studied. Data mostly reported MSI status which in early-onset had higher rate of MSI-H [7]. Data on other genes mutation are scant, some reporting the lower rate of KRAS and BRAF mutation among this group [5]. There is controversy over survival differences between younger and older age groups; one large review reported 10-year overall survival rates of 38.6% and 56.9% for AYA and OA patients [8]. All literature points to the delay in diagnosis as the reason for worse clinical outcome in younger patients.

Here we report a case of a 20-year-old man who was diagnosed with stage IVB signet-ring cell adenocarcinoma of the colon (T4 N2 M1, with peritoneal carcinomatosis). We review the literature on this subtype and provide direction for future research efforts.

## 2. Case Report

A 20-year-old white man with a medical history of asthma and attention deficit hyperactive disorder presented with new-onset right lower quadrant abdominal pain with associated nausea and vomiting in September 2012. He had no significant family history of illness. He underwent colonoscopy, which showed a circumferential malignant-appearing mass at the hepatic flexure. The scope could not pass through the mass. Biopsy revealed poorly differentiated signet-ring cell adenocarcinoma. A computed tomography (CT) scan of the abdomen and pelvis showed irregular area of thickening of the bowel wall at the hepatic flexure with associated adenopathy, without evidence of distant metastatic disease. The patient's carcinoembryonic antigen level was within the normal range.

**Figure 1:**
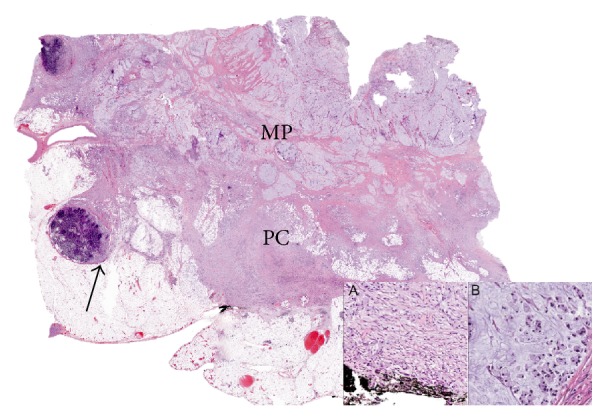
Signet-ring cell adenocarcinoma infiltrating through the muscularis propria (MP) into pericolic adipose tissue (PC) and involving regional lymph nodes (arrow). The signet-ring cells are seen infiltrating through tissue and extending to the serosal surface (A) and are seen floating in mucin (B).

The patient subsequently underwent exploratory laparotomy. Intraoperative findings revealed an island of peritoneal nodules adherent to the omentum and studding the peritoneum along the right pericolic gutter. Therefore, extended right hemicolectomy with total omentectomy and partial peritonectomy was performed, and pathologic analysis of the surgical specimens confirmed the diagnosis of poorly differentiated signet-ring cell adenocarcinoma at the hepatic flexure with penetration to the serosal surface (Figure 1). Seven of 44 regional lymph nodes were involved, and a peritonectomy specimen confirmed carcinomatosis. Five hamartomatous polyps at the ascending colon were also reported. Therefore, the patient was diagnosed with stage IVB colon cancer (T4 N2 M1, with peritoneal carcinomatosis).

A next-generation sequencing-based analysis of 46 genes showed* TP53* and* SMAD4* gene mutations, but* STK11*,* PTEN*,* KRAS*,* NRAS*, and* BRAF* genes were wild-type. Mutations were detected in codon 361, exon 9 of* SMAD4* gene, and in codon 273, exon 8 of* TP53* gene. Because of the hamartomatous polyps and early age of onset, germline sequencing of* BMPR1* and* SMAD4* was performed, but this did not demonstrate any mutations. Microsatellite instability testing by polymerase chain reaction and immunohistochemistry demonstrated microsatellite stability.

Given the carcinomatosis, despite the lack of radiographically evaluable disease, the patient was treated with the FOLFOX regimen (folinic acid, fluorouracil, oxaliplatin) and bevacizumab. A restaging evaluation with chest and abdominal CT after 11 cycles of the treatment showed progression of the peritoneal metastases. The treatment was then changed to the FOLFIRI regimen (folinic acid, fluorouracil, irinotecan) plus cetuximab. Restaging studies after 6 cycles showed progressive disease in the peritoneum. The treatment was changed to regorafenib. Approximately 6 weeks later, in December 2013, the patient developed a bowel obstruction in spite of treatment. The obstruction was thought to be localized, and a palliative resection of a portion of the small bowel was planned. However, evidence of disease progression on restaging CT ruled out surgical options. The patient's performance status declined afterward, and he was transferred to supportive care. The patient was placed on PCA hydromorphone and was discharged to hospice care.

## 3. Discussion

Classical adenocarcinoma is the most common histologic subtype of CRC, whereas mucinous subtype accounts for 10–20% and signet-ring cell subtype for 0.9–4% of cases of CRC [20]. However, mucinous differentiation can be found in 6.5–30.6% and signet-ring histology can be found in 3–33% in young onset patients [5, 21]. Although only 10% of people diagnosed with CRC are below 50 years of age, incidence in this age group has seen a rise [22]. Between 1992 and 2005, the incidence of CRC in young men and women aged 20–49 years increased at annual rates of 1.5% and 1.6%, respectively [1]. The histopathologic characteristics and clinical behavior of AYA CRC patients have been shown to be distinct from those of CRC in older adults where AYA CRC patients have higher rates of hereditary CRC syndromes and inflammatory bowel disease and a lower rate of polyps. Additionally, they are more likely to present with stage III or IV disease, angiolymphatic invasion, perineural invasion, signet-ring cell adenocarcinoma, and distal tumors location [5, 23].

There were few literatures on clinical outcome in AYA CRC patients. Most of them included hereditary CRC which might confound the true outcome leading to the conflict results. A large population based analysis with SEER data reported an equivalent or better survival rate compared with older patients [24]; however, some reported no significance in OS or recurrence-free survival in two groups [5], while some demonstrated poor outcomes in early-onset group [25–27]. More recently, Fu et al. reported 10-year OS lower in CRC patients under 35 years compared with those over 35 years (38.6% versus 56.9%, *P* < 0.001) in univariate analysis [8]. However, this effect was not statistically significant in multivariate analysis after correcting for tumor stage (*P* = 0.053).

A study of CRC patients from SEER 9 Registries Data showed that the incidence of signet-ring cell histology was 3.2% in patients <40 years of age and 1.4% in patients aged between 40 and 50 years [20]. This histologic subtype is distinguished from typical adenocarcinoma by the excess amount of mucin within the tumor. Occasionally, mucin accumulates intracellularly in these tumors and displaces the nucleus, which results in signet-ring appearance of the cells. Signet-ring cell carcinoma is defined by the presence of this distinctive morphologic characteristic in greater than 50% of the tumor. Signet-ring cell carcinoma (SRCC) has been associated with young age, advanced tumor stage at presentation, distant lymph node metastasis, and distinct molecular patterns such as a high rate of microsatellite instability and* BRAF* gene mutations and a low rate of* KRAS* gene mutations [28–31]. Chew et al. and Börger et al. demonstrated that SRCC was associated with higher T stages, more frequent nodal invasion, and significantly poorer prognosis than adenocarcinomas and mucinous carcinomas [32, 33]. A higher degree of microsatellite instability is associated with a better outcome. Our patient demonstrated microsatellite stability; hence the stage IV presentation was unsurprising.

The peritoneal surface is a common site of metastasis for CRC, occurring in 13% of patients. Synchronous peritoneal metastases from CRC are most common among young patients and those with advanced T stage, mucinous adenocarcinoma, right-sided tumors, and tumors that are poorly differentiated [34, 35]. SRCC most frequently metastasizes to the peritoneum (38.7%) and only rarely to the liver or lung (2.9%), which is opposite to the trend seen in metastasis of typical adenocarcinomas [14, 36–38]. The propensity of SRCC to peritoneal seeding has been explained by the excessive production of mucus under pressure, which allows tumor cells to traverse the bowel wall to enter the peritoneal cavity [39]. Additionally, SRCC shows significantly reduced expression of cell adhesion molecule E-cadherin, which forms E-cadherin-catenin complexes that maintain epithelial cell polarity. Loss of E-cadherin leads to epithelial dedifferentiation and acquisition of a motile or infiltrative phenotype [36, 40]. Other sites of metastasis that are more common to SRCC than adenocarcinoma include the heart, bone, pancreas, and skin [39]. The lower rate of liver/lung metastases in SRCC is likely responsible for the much lower success rate of curative resection in SRCC patients [14]. Chemotherapy in patients with peritoneal metastases may not produce the same results as in patients with hematogenous metastases, leading to poor outcomes in patients with advanced disease, who have a median survival duration of about 6 months [34, 41]. All cases of SRCC with peritoneal dissemination have reported rapid disease progression in spite of both systemic and intraperitoneal chemotherapy [42]. A recent review concluded that, owing to its aggressiveness, the presence of signet-ring histology may be a relative contraindication for cytoreductive surgery with hyperthermic intraperitoneal chemotherapy (CRS + HIPEC). This indicates at present a therapeutic dead-end for SRCC patients with peritoneal carcinomatosis and a significant area for future research [43].

Information is scant on signet-ring cell carcinoma, and fewer than 20 cases have been discussed in the literature, as shown in Table 1.

In our patient, given the early onset of disease, a predisposing genetic syndrome was considered. The microsatellite stability of the tumor significantly reduced the likelihood of Lynch syndrome, which is the most common cause of hereditary CRC. Because multiple hamartomatous polyps were identified in our patient, hereditary hamartomatous polyp diseases, which include juvenile polyposis syndrome, Peutz-Jeghers syndrome, and Cowden disease, were considered. The* STK11* and* PTEN* genes were wild-type, and no clinical characteristics suggested Peutz-Jeghers syndrome or Cowden disease. Although the* SMAD4* gene was mutated in the tumor, it was not mutated in the blood sample, and* BMPR1* gene mutations were not found either. Both of these gene mutations can be identified in 20% of patients with autosomal-dominantly inherited familial juvenile polyposis; therefore, this patient was unlikely to have juvenile polyposis syndrome [44]. Moreover, the patient had no juvenile polyps in any other part of the gastrointestinal tract and no family history of juvenile polyposis syndrome. However, we cannot exclude the possibility of these diseases completely.

## 4. Conclusion

We have reported a rare, aggressive case of early-onset signet-ring cell adenocarcinoma with peritoneal metastasis. Owing to the relative rarity of the signet-ring cell histologic subtype, molecular and cellular characteristics are just beginning to accumulate within our existing knowledge. This case study highlights the need for additional study of the biological and molecular landscape of this disease.

## Figures and Tables

**Table 1:** Cases of signet-ring cell carcinoma (SRCC) of the colon reported in the literature.

Age, years/sex	History of inflammatory bowel disease	Initial diagnostic test	Polyps identified	Positive markers	Location in colon	Stage at diagnosis	Metastasis	Chemotherapy	Survival	Citation
9/M	No	CT abdomen/pelvis	Unspecified	Unspecified	Transverse colon, proximal to splenic flexure	Unspecified	Initially not identified, identified after 7 cycles of chemotherapy	Unspecified	One year + 2 weeks	Yang et al. [9]

36/F	No	Unspecified	No	MLH1, PMS2, MSH2, MSH6; microsatellite stability according to PCR	Cecum	IIA (T3 N0 M0)	No	Adjuvant 5-fluorouracil-based therapy with oral capecitabine	N/A	Park et al. [10]

73/M	No	Screening colonoscopy	No	MUC2, MUC5AC positive; MUC6 focally positive; MUC1 negative	Cecum	Unspecified	No	Unspecified	At 26-month postoperative follow-up, patient is in good health	Ohnita et al. [11]

67/M	No	Surveillance colonoscopy for previously resected non-SRCC gastric cancer	Focal cancer in an adenoma, completely resected one year before	Tumor positive for MSH, negative for MLH1	Transverse colon near splenic flexure	Unspecified	No	Unspecified	Alive at 5-year postoperative follow-up	Fu et al. [12]

19/M	Suspected because of inflammation and edema around the ileal wall	Colonoscopy/gastroscopy because of suspected bowel/peritoneal disease observed on computed tomography scan	No	Unspecified	Sigmoid colon	Unspecified	SRCC cells seen in abdominal fluid	FOLFOX-6 with bevacizumab	Still under treatment	Pamukçu et al. [13]

31/M					Rectosigmoid			Unspecified	Unspecified	Tung et al. [14]

17/M	No	Ultrasound showing aperistalsis of a segment of the bowel wall, followed by computed tomography	No	Unspecified	Ascending colon	T4 N2 M1	Unspecified	FOLFOX-6	Alive at 1-year postoperative follow-up	Marone et al. [15]

13/M	No	Lower gastrointestinal barium study and colonofibroscopy	No	Unspecified	Ascending colon	Unspecified	Unspecified	5-fluorouracil, mitomycin, levamisole	Alive at 1-year postoperative follow-up	Ko et al. [16]

25/M	No	Workup for bladder complaints	No	Unspecified	“Rectal diverticulum” (pulled through sigmoid colon), 2 cm above the anus	Unspecified	Infiltrating the bladder and abdominal wall	Unspecified	Alive at 7-month postoperative follow-up	Posey et al. [17]

6/M	No		Yes	Unspecified	Polyp in descending colon	Unspecified	Unspecified	Unspecified	Unspecified	Hamazaki et al. [18]

22/F	No	Double-contrast barium radiography	No	Unspecified	Descending colon	Dukes B carcinoma	No	Unspecified	Followed up for 3 years without recurrence	Nakata et al. [19]

N/A: not applicable.
